# c-Jun N-terminal kinase activation by nitrobenzoxadiazoles leads to late-stage autophagy inhibition

**DOI:** 10.1186/s12967-016-0796-x

**Published:** 2016-02-04

**Authors:** Camilla Palumbo, Anastasia De Luca, Nicola Rosato, Mariantonietta Forgione, Dante Rotili, Anna Maria Caccuri

**Affiliations:** Department of Clinical Sciences and Translational Medicine, University of Rome Tor Vergata, Via Montpellier 1, 00133 Rome, Italy; Department of Experimental Medicine and Surgery, University of Rome Tor Vergata, Via Montpellier 1, 00133 Rome, Italy; The NAST Centre for Nanoscience and Nanotechnology and Innovative Instrumentation, University of Rome Tor Vergata, Via della Ricerca Scientifica 1, 00133 Rome, Italy; Department of Drug Chemistry and Technologies, Sapienza University of Rome, Piazzale Aldo Moro 5, 00185 Rome, Italy; Center for Life Nano Science@Sapienza, Italian Institute of Technology, Viale Regina Elena 291, 00161 Rome, Italy

**Keywords:** Nitrobenzoxadiazoles, Glutathione transferase, c-Jun N-terminal kinase, Autophagy, Osteosarcoma

## Abstract

**Background:**

Nitrobenzoxadiazole derivatives (NBDs), including NBDHEX and the recently developed MC3181, are promising anticancer agents able to target glutathione transferase and inhibit both its catalytic activity and ability to sequester TNF-receptor associated factor 2 (TRAF2) and c-Jun N-terminal kinase (JNK). NBDs have been shown to impair the growth and survival of a broad-spectrum of tumor types, in vitro and in vivo. Herein, we evaluated the effects of the new compound MC3181 on U-2OS osteosarcoma cells and investigated the impact of both NBDHEX and MC3181 on autophagy.

**Methods:**

Cell viability was evaluated by sulforhodamine B assay. The dissociation of the TRAF2-GSTP1-1 complex was detected by proximity ligation assay, while the phospho-activation of JNK was assessed by western blotting. The effects of NBDs on autophagy were evaluated by GFP-LC3 puncta formation, western blotting for LC3-II and p62, and LC3 turnover assay in the presence of bafilomycin A1. The role of JNK in the reduction of autophagic flux caused by NBDs was investigated using JNK1 shRNA-transfected cells. Fluorogenic caspase activity assay and flow cytometric analysis of DNA content were used to determine the cytotoxic effects of NBDs on JNK1-silenced cells.

**Results:**

Similar to NBDHEX, MC3181 reduced viability and activated TRAF2/JNK signaling in U-2OS cells. Moreover, NBDs induced the accumulation of autophagic vesicles and LC3-II while reducing both basal and nutritional stress-induced autophagic flux. Furthermore, increased levels of both LC3-II and the autophagy selective substrate p62 were observed in different tumor cell lines treated with NBDs, the concurrent increase of these markers being consistent with an impairment of autophagosome clearance. Autophagy inhibition by NBDs required JNK activity: NBDs caused autophagy inhibition and caspase-3 activation in JNK-positive U-2OS, but no autophagic flux inhibition or caspase-3 activation in JNK-silenced cells.

**Conclusions:**

Our demonstration that NBDs can act as late-phase autophagy inhibitors opens new opportunities to fully exploit their therapeutic potential. This may not rely solely on their effectiveness in inducing cell cycle arrest and apoptosis, but also on their ability to weaken the capacity of tumor cells to endure stress conditions via autophagy. In addition, this study provides evidence that JNK can participate in impairing autophagy.

## Background

NBDHEX (6-((7-nitrobenzo[c][1,2,5]oxadiazol-4-yl)thio)hexan-1-ol) is the leading compound of a class of nitrobenzoxadiazole derivatives (NBDs) with promising anticancer properties. Indeed, by targeting glutathione transferases (GSTs) these compounds have been shown to impair growth and survival of cancer cells at multiple levels [1–5]. First, NBDs act as strong inhibitors of GSTs catalytic activity; accordingly, they can hinder the GST-mediated conjugation of several electrophilic anticancer drugs to reduced glutathione (GSH), which in turn would result in drug detoxification and extrusion from the cell via specific export pumps [1, 6–9]. Moreover, NBDHEX is not a substrate of P-glycoprotein (P-gp) and multidrug resistance-associated protein 1 (MRP1) transporters, so that it accumulates in tumor cells, overcoming another major mechanism of cancer cell chemoresistance [10–12]. Secondly, NBDs are able to disrupt the interaction between the GST isoform GSTP1-1 and key signaling effectors involved in the regulation of cell survival and proliferation, namely the adaptor protein TNF-Receptor associated factor 2 (TRAF2) and the c-Jun N-terminal kinase (JNK) [2, 3, 13, 14]. The NBDs-induced release of TRAF2 from the complex with GSTP1-1 leads to the activation of the apoptosis signal-regulating kinase (ASK1), which in turn activates both p38 and JNK mitogen-activated protein kinase (MAPK) signaling pathways [3, 8]. As a result, p38 causes cell cycle arrest, while JNK promotes apoptosis, the activation of this MAPK pro-apoptotic pathway being further sustained by the NBDs-induced release of JNK from the complex with GSTP1-1 [3, 9]. Therefore, NBDs can exert antitumor effects either per se or by potentiating the efficacy of conventional anticancer drugs whose action relies on the activation of these MAPK pathways [9].

In fact, NBDHEX has shown a broad-spectrum of activity against cancer cells of different origins, including osteosarcoma, Ewing’s sarcoma, melanoma, mesothelioma, lung and hepatic carcinoma and different types of leukemia, either alone or in combination with antitumor drugs such as cisplatin, doxorubicin, vincristine, and temozolomide [2, 3, 10, 15–18]. Moreover, in in vivo studies performed on various tumor types xenografted in mice, NBDHEX proved to be effective in reducing both cancer growth and metastatic spread and was well tolerated [17, 19, 20].

Besides NBDHEX, the most promising compound among the NBDs is the recently developed water-soluble analogue MC3181 (2-(2-(2-((7-nitrobenzo[c][1,2,5]oxadiazol-4-yl)thio)ethoxy)ethoxy)ethanol) [5, 21]. This compound bears two oxygen atoms within the alkyl chain bound at the C4 position of the NBD scaffold, resulting in a more than 50-fold increase in aqueous solubility compared to NBDHEX [5].

Given the encouraging results obtained with NBDHEX on osteosarcoma models [3, 15, 20], we performed experiments initially aimed at evaluating the activity of the new compound MC3181 on U-2OS human osteosarcoma cells cultured in vitro. Treatment with either MC3181 or the parent compound NBDHEX caused the accumulation of cytoplasmic vacuoles in U-2OS cells and prompted us to investigate the possible effects of these NBDs on autophagy. We here demonstrate that NBDs cause late-stage autophagy inhibition via the activation of JNK.

## Methods

### Cell culture and treatments

U-2OS human osteosarcoma cells were purchased from the American Type Culture Collection (ATCC) and grown in IMDM supplemented with 10 % FBS, 2 mM l-glutamine, 100 U/ml penicillin and 100 µg/ml streptomycin (EuroClone, Milan, Italy). Experiments were also performed with MM-B1 biphasic malignant mesothelioma cells [18] and with the HT-29 and COLO 205 colorectal adenocarcinoma and MCF-7 breast adenocarcinoma cells of the NCI-60 cell line panel. MCF-7, COLO 205 and HT-29 cells were grown in RPMI 1640, and MM-B1 in DMEM (EuroClone), both media being supplemented as above. NBDHEX and MC3181 were synthesized as previously reported [1, 5]. For cell treatments NBDs were dissolved in DMSO and diluted in cell medium, with the final DMSO concentration never exceeding 0.01 % (v/v); control cultures received an equivalent amount of DMSO vehicle. The late-stage autophagy inhibitor chloroquine diphosphate (CQ) was purchased from Sigma-Aldrich (Milan, Italy).

### Cell viability studies

Cells (7–35 × 10^3^, depending on the cell line) were seeded in triplicate in 96-well plates and cultured for 24 h, after which they were exposed to increasing concentrations (0.1–50 μM) of MC3181 and NBDHEX. Cell viability was quantified after 48 h of treatment by the sulforhodamine B (SRB) assay [22]. The concentrations of drug required for 50 % inhibition of cell viability (IC_50_) were determined from dose–response curves.

### Proximity ligation assay (PLA)

PLA and confocal laser scanning microscopy analysis of TRAF2-GSTP1-1 complexes were performed as previously described [14]. Briefly, formaldehyde-fixed and methanol-permeabilized U-2OS cells were incubated with a mouse anti-GSTP1-1 and a rabbit anti-TRAF2 antibody (Cell Signaling Technology, Beverly, MA, USA) followed by Duolink PLA Rabbit MINUS and PLA Mouse PLUS proximity probes (Olink Biosciences, Uppsala, Sweden). Proximity ligation was performed using the Duolink Far-Red in situ detection reagent kit (Olink Bioscences), according to the manufacturer’s protocol. At the end of the procedure each TRAF2-GSTP1-1 complex generated a fluorescent red spot. DAPI was used to counterstain cell nuclei. Fluorescence was detected using a Fluoview 1000 Olympus system equipped with an Olympus IX-81 inverted microscope, as previously detailed [14]. The BlobFinder software (Olink Bioscience) was used for PLA image analysis. Fluorescent signals from rolling circle amplification products were defined and counted per cell.

### Western blotting

Western blot analysis was performed using the following primary antibodies: anti-phospho-JNK (Thr183/Tyr185) and anti-JNK1 (Cell Signaling Technology); anti-LC3 (Novus Biologicals, Littleton, CO, USA) [23]; anti-p62/SQSTM1 (Santa Cruz Biotechnology, Santa Cruz, CA, USA) [24]; anti-β-actin (Sigma-Aldrich). The immune complexes were visualized using an enhanced chemiluminescence detection system (Pierce, Rockford, IL, USA). Densitometric analysis of autoradiographic bands was performed using the NIH Image J software (National Institutes of Health, Bethesda, MD, USA).

### GFP-LC3 transfection

U-2OS cells were transfected with the pEGFP-LC3 plasmid [25], kindly provided by Professor Yoshimori (Department of Genetics, Osaka University, Osaka, Japan), using the calcium phosphate/BES method [26]. After about 2 weeks of selection in G418-containing medium (400 μg/ml; Sigma-Aldrich) and one additional week of growth in medium without G418, the entire population of transfected cells was used for GFP-LC3 puncta formation assays [25, 27]. The formation of puncta was evaluated under an Olympus BX50 fluorescence microscope equipped with a digital camera.

### Assessment of autophagic flux by LC3 turnover assay

U-2OS cells were seeded in duplicate at a density of 2 × 10^4^ cells/cm^2^, cultured for 24 h, treated with NBDs in medium containing 10 or 0.2 % FBS for an additional 24 h, and then lysed in triple-detergent lysis buffer. Three hours before lysis, one of the duplicate cultures was incubated with 100 nmol/l of the late-stage autophagy inhibitor bafilomycin A1 (BAF; Sigma-Aldrich), while the other was incubated with the BAF vehicle DMSO. Cell lysates were analyzed by western blotting with anti-LC3 and anti-β-actin antibodies. After densitometric analysis of autoradiographic bands, the autophagic flux indexes of NBDs-treated and untreated cultures were calculated as the difference in LC3-II/actin ratios between samples plus and minus BAF [28–30]. The autophagic flux index of NBD-treated cultures was expressed in proportion to that of untreated cultures, which was arbitrarily set equal to 1.

### JNK1 RNA interference

Vectors for JNK1 RNA interference were purchased from OriGene Technologies, Rockville, MD, USA. U-2OS cells were calcium phosphate/BES-transfected [26] with pGFP-V-RS vectors carrying either a 29-mer short hairpin RNA (shRNA) for human JNK1 (MAPK8; vector TG320484) or a 29-mer scrambled shRNA cassette (vector TR30013). Transfected cells were selected with puromycin (1 μg/ml; Sigma-Aldrich) and individual clones were expanded and characterized for JNK1 protein expression levels. One JNK1 shRNA clone and one scrambled shRNA clone were chosen for functional studies, which were performed using cells grown for about 1 week in the absence of puromycin.

### Caspase activity assay

Caspase activity was measured as previously described [2]. Briefly, lysates from 1 × 10^6^ U-2OS cells were assayed using the fluorogenic caspase-3 substrate *N*-acetyl-Asp-Glu-Val-Asp-7-amido-4-trifluoromethylcoumarin (Ac-DEVD-AFC, Sigma-Aldrich). Ac-DEVD-AFC cleavage was measured with a fluorometer equipped with a 400-nm excitation filter and a 505-nm emission filter. The rate of fluorescence increase (ΔF/min) was calculated and results were expressed as ΔF/min × 10^6^ cells.

### Analysis of cellular DNA content by flow cytometry

U-2OS cells were fixed with 70 % ethanol, stained with 50 mg/ml propidium iodide in a buffer containing 10 mg/ml RNase and 1 % Triton X-100, and analyzed with a FACSCalibur flow cytometer (BD Bioscences, San Jose, CA, USA). Data analysis was performed using the FlowJo 8.8.6 software (Tree Star, Ashland, OR, USA).

### Statistical analysis

The experiments were repeated at least three times. Statistical analysis was performed by Student’s two-tailed *t* tests, with a significance threshold set at p < 0.05.

## Results

### Similar to the parent compound NBDHEX, MC3181 reduces viability and activates TRAF2/JNK signaling in osteosarcoma cells

**Fig. 1:**
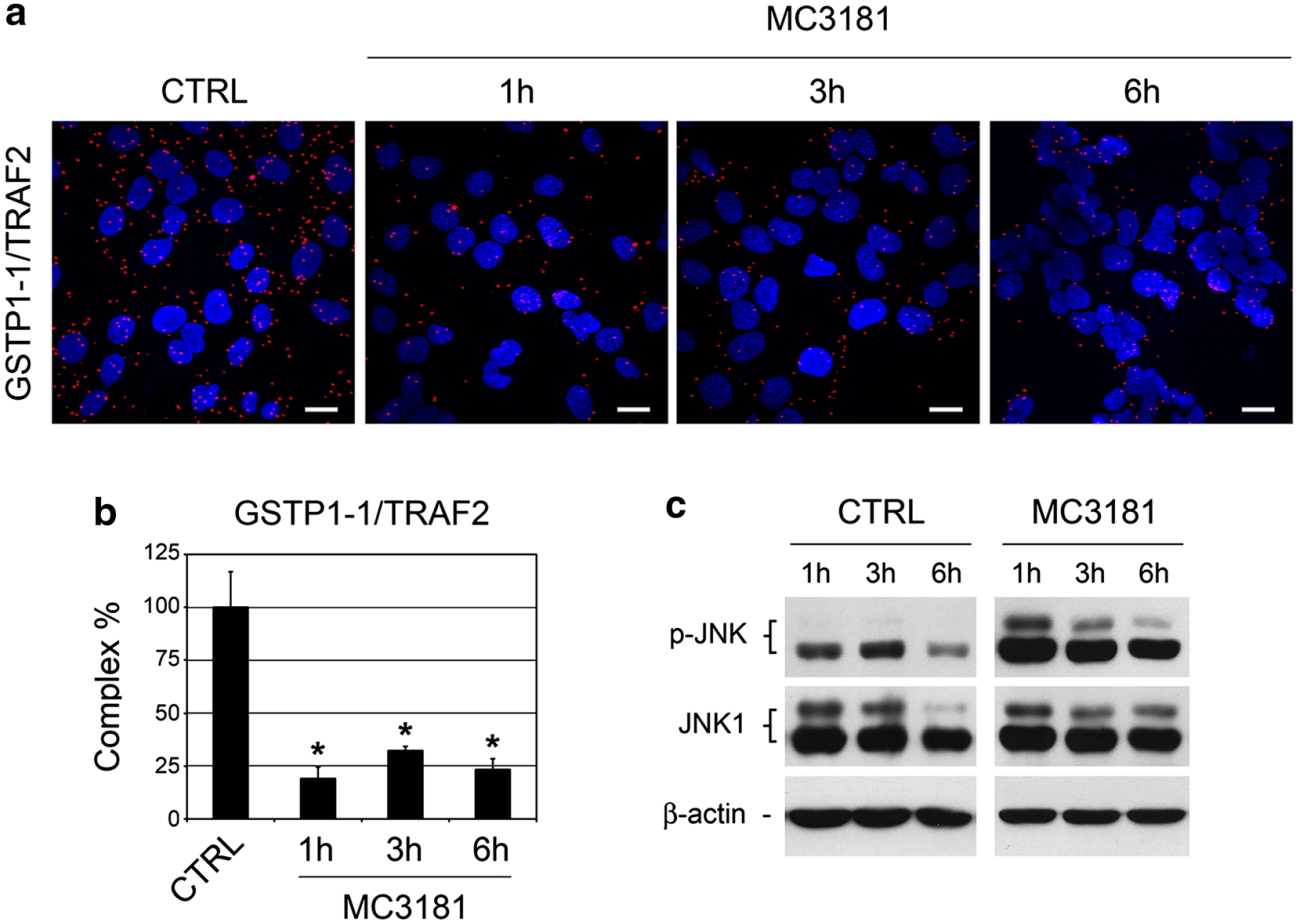
Effects of MC3181 on TRAF2/JNK signaling in U-2OS cells. **a** Confocal microscopy for the in situ detection of the TRAF2-GSTP1-1 complex in cells treated with vehicle (CTRL) or 5 μM MC3181 (i.e. a concentration about five times the IC_50_ value) for the indicated times. The TRAF2-GSTP1-1 complex was visualized by PLA, which generates fluorescent dots (*red*) when the two proteins are in close proximity. DAPI (*blue*) was used to counterstain cell nuclei. *Scale bar* 10 μM. **b** Amount per cell of TRAF2-GSTP1-1 complex as calculated from the analysis of PLA images (mean ± SEM, n ≥ 3); *p < 0.05. **c** Western blot analysis of JNK phospho-activation in cells treated with vehicle (CTRL) or 5 μM MC3181 for the indicated times. The filter was probed with anti-phospho-JNK (p-JNK) and anti-JNK1 antibodies; β-actin was used to ensure equal loading and transfer of samples

We first evaluated the effects of MC3181 on viability of U-2OS human osteosarcoma cells. Similar to NBDHEX, MC3181 reduced the viability of U-2OS cells at concentrations in the low micromolar range, the calculated IC_50_ values after a 48-h treatment being 0.9 μM for NBDHEX and 1.1 μM for MC3181 (Table 1). Our group has previously demonstrated that NBDs trigger the activation of JNK signaling in tumor cell lines of various origins [2, 5, 11, 18]. In particular, we already documented that NBDHEX dissociates the complex between GSTP1-1 and TRAF2, and induces the activation of JNK in U-2OS cells [3, 14]. By monitoring the TRAF2-GSTP1-1 interaction in situ by PLA (Fig. 1a) and the phospho-activation of JNK by western blotting (Fig. 1b) over a 6-h time-course, we here show that MC3181 shares the property of the parent compound to promote TRAF2/JNK signaling activation in U-2OS cells. Of note, the decrease in the amount of TRAF2-GSTP1-1 complex observed in MC3181-treated cells was not related to variations in the levels of the individual proteins (not shown).

**Table 1:** NBDHEX and MC3181 IC_50_ values for U-2OS and tumor cell lines of different histotypes

Cell line	NBDHEX (μM)	MC3181 (μM)
U-2OS	0.9 ± 0.1	1.1 ± 0.1
MM-B1	1.3 ± 0.1	1.6 ± 0.2
MCF-7	1.6 ± 0.1	1.5 ± 0.1
COLO 205	5.3 ± 0.4	6.5 ± 0.2
HT-29	11.0 ± 0.4	15.8 ± 0.8

IC_50_ values (mean ± SEM, n ≥ 3) after 48 h of treatment with NBDs, as determined from cell viability dose–response curves

### MC3181 and NBDHEX induce accumulation of autophagosomes

**Fig. 2:**
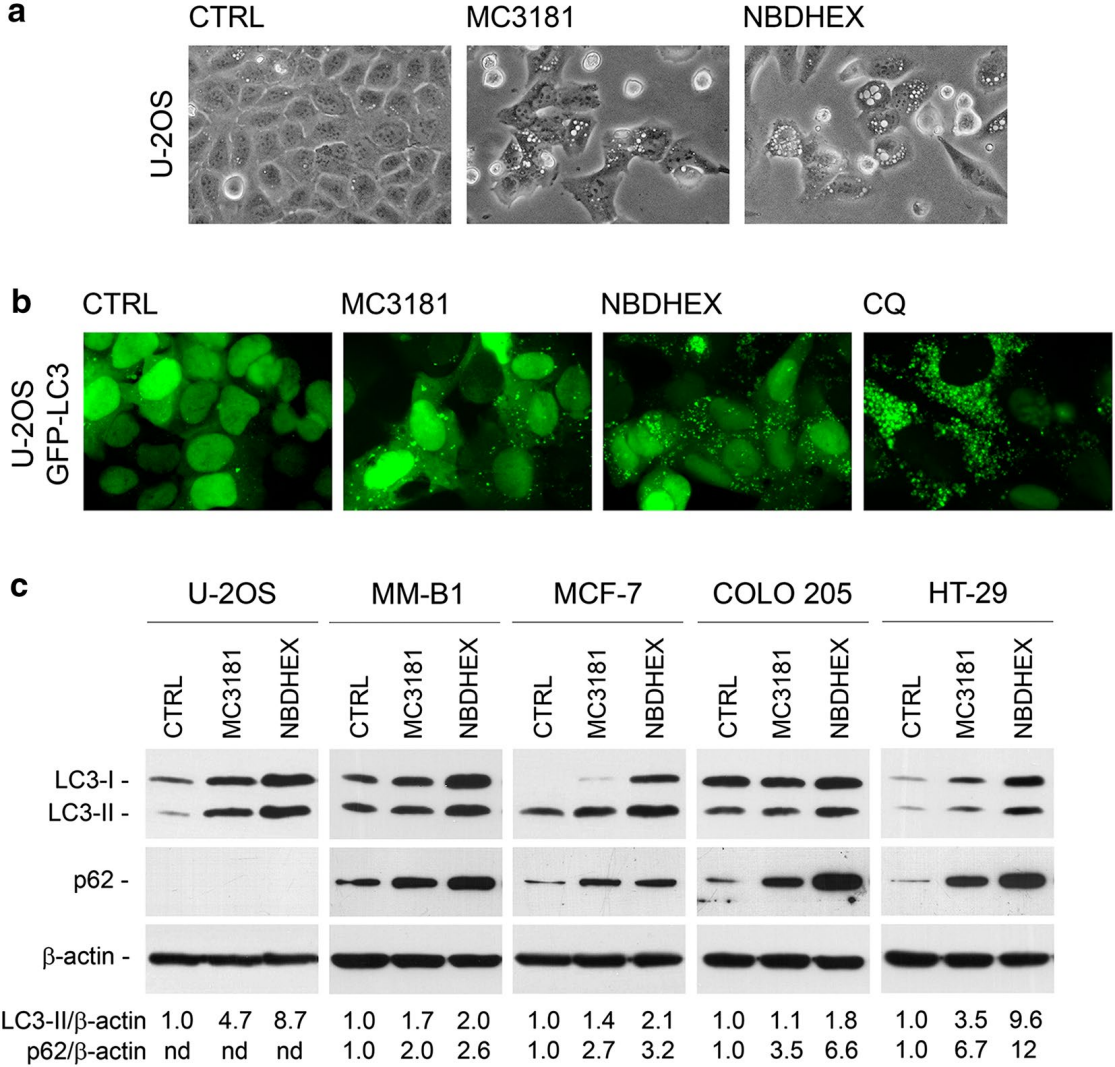
MC3181 and NBDHEX induce accumulation of both autophagic vesicles and the selective autophagy substrate p62. **a** Morphology of U-2OS cells treated with vehicle (CTRL), 5 μM MC3181 or 5 µM NBDHEX for 48 h, viewed under an Olympus IX50 inverted microscope equipped with a digital camera. Original magnification ×200. **b** Puncta formation assay on U-2OS GFP-LC3 cells treated with vehicle (CTRL), 5 μM NBDs or 10 μM CQ for 48 h. Cells were photographed using a fluorescence microscope equipped with a digital camera. Original magnification ×400. **c** Immunoblot analysis for LC3-II and p62 in lysates from U-2OS, MM-B1, MCF-7, COLO 205 and HT-29 cells treated with vehicle (CTRL), 5 μM MC3181 or 5 µM NBDHEX for 48 h. β-Actin was used to ensure equal loading and transfer of samples. LC3-II/β-actin and p62/β-actin densitometric ratios are also reported: those of MC3181- and NBDHEX-treated samples were expressed in proportion to those of CTRL samples, which were arbitrarily set, equal to 1

U-2OS cultures treated with either MC3181 or NBDHEX were characterized by the presence of both dead cells and cells showing a notable degree of cytoplasmic vacuolization, suggestive of the accumulation of autophagic vesicles (Fig. 2a). The hypothesis that NBDs could induce an accumulation of autophagosomes was confirmed by puncta formation assays performed using U-2OS cells transfected with a GFP-LC3 expression vector (Fig. 2b). Transfected cells left untreated displayed variable levels of GFP-LC3 fluorescence, diffuse in appearance and mainly localized to the nuclei; conversely, in transfected cells treated with either MC3181 or NBDHEX, GFP-LC3 was partly redistributed into numerous cytoplasmic puncta representing bona fide autophagosomes [27, 31]. Moreover, GFP-LC3 translocation from the nucleus to cytoplasmic dots appeared nearly complete in cells treated with the late-stage autophagy inhibitor CQ (Fig. 2b), used as positive control for autophagic vesicles accumulation [29].

### MC3181 and NBDHEX induce accumulation of both LC3-II and the selective autophagy substrate p62

The number of autophagosomes is known to correlate with the amount of LC3-II protein, which is formed by lipidation of the cytosolic LC3-I protein [28]. In line with the above reported findings, wild type U-2OS cells treated with NBDs for 48 h had increased amounts of the autophagosome-associated LC3-II protein as assessed by western blot analysis (Fig. 2c) [28]. Moreover, following NBDs treatment variably increased levels of LC3-II were also observed in a panel of tumor cell lines (including malignant mesothelioma, breast and colon carcinoma) characterized by different degrees of sensitivity to the cytostatic/cytotoxic effects of these compounds (Fig. 2c; Table 1).

The accumulation of autophagic vesicles and LC3-II induced by NBDs could be due to an increased generation of autophagosomes, to a decreased autophagosomal maturation/degradation or to a combination of both processes [27, 28]. In order to understand whether the net effect of NBDs would be an enhanced or reduced clearance of autophagy substrates, we performed western blot analysis for p62 (SQSTM1), one of the specific substrates that are selectively degraded through the autophagy-lysosomal pathway [28]. In U-2OS cells p62 levels were very low to undetectable, in line with previous reports [32, 33]. On the other hand, in MM-B1, MCF-7, COLO 205 and HT-29 cells p62 was detectable in basal conditions and found to increase upon treatment with either NBD compound (Fig. 2c). The concurrent increase of p62 and LC3-II in these cell types indicates that NBDs cause an accumulation of autophagic vesicles at least in part via an impairment of autophagosome maturation/degradation, in this respect behaving as late-stage autophagy inhibitors [27, 28]. Based on the increase of both LC3-II and p62 levels, NBDHEX demonstrated a higher efficacy in reducing autophagosome clearance as compared to MC3181 (Fig. 2c).

### NBDs impair both basal and nutritional stress-induced autophagic flux

**Fig. 3:**
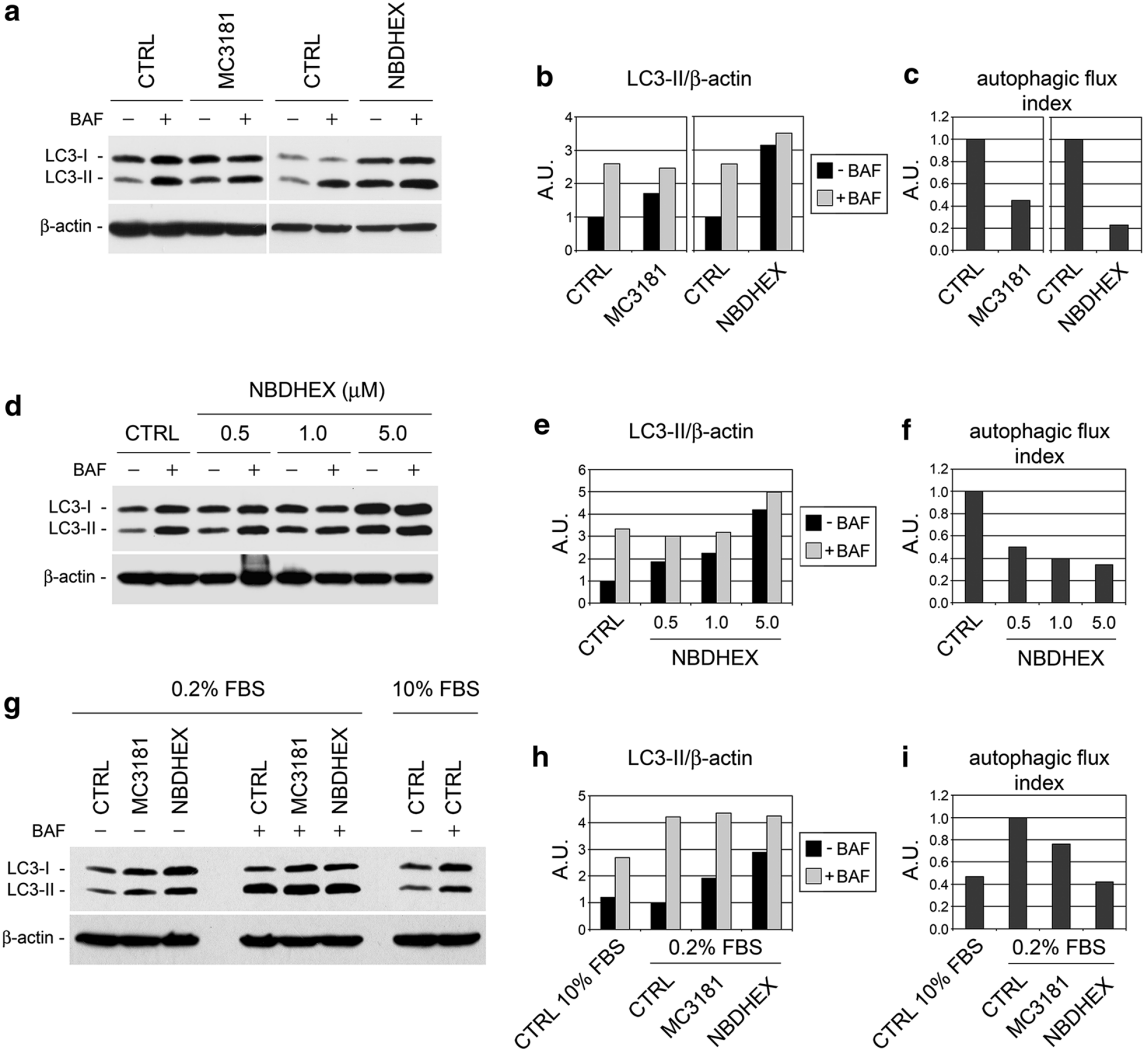
Effect of NBDs on the autophagic flux of U-2OS cells. **a** LC3 turnover assays in basal conditions. Lysates from cells treated with vehicle (CTRL), 5 μM MC3181 or 5 μM NBDHEX for 24 h, incubated or not with 100 nmol/l BAF for 3 h before lysis, were subjected to western blot for LC3-II and β-actin. **b** LC3-II/β-actin ratios determined from the densitometric analysis of the autoradiograms shown in **a** expressed in arbitrary units (a.u.). **c** Autophagic flux indexes calculated from the data illustrated in **b** as the difference in LC3-II/actin ratios between samples plus and minus BAF, expressed in arbitrary units (a.u.). **d** Dose–response effect of NBDHEX on basal autophagic flux. Lysates from cells treated with vehicle (CTRL) or 0.5–5 μM NBDHEX for 24 h, incubated or not with 100 nmol/l BAF for 3 h before lysis, were subjected to western blot for LC3-II and β-actin. **e** LC3-II/β-actin ratios determined from the densitometric analysis of the autoradiograms shown in **d**. **f** Autophagic flux indexes calculated from the data illustrated in **e** as the difference in LC3-II/actin ratios between samples plus and minus BAF. **g** LC3 turnover assays in nutritional stress conditions. Lysates from cells treated with vehicle (CTRL) or 5 μM NBDs in the presence of 0.2 % FBS for 24 h, incubated or not with 100 nmol/l BAF for 3 h before lysis, were subjected to western blot for LC3-II and β-actin. Lysates from cells grown for 24 h in the presence of 10 % FBS, incubated or not with BAF as above, were included to assess basal flux levels. **h** LC3-II/β-actin ratios determined from the densitometric analysis of the autoradiograms shown in **g**. **i** Autophagic flux indexes calculated from the data illustrated in **h** as the difference in LC3-II/actin ratios between samples plus and minus BAF

The term “autophagic flux” refers to the dynamic equilibrium between autophagosome formation and clearance by lysosomes. In other words, autophagic flux is the rate at which material is actually cleared from the cell by autophagy. Based on the observation that LC3-II is degraded in autophagolysosomes, autophagic flux can be quantified by measuring LC3 turnover [27, 28]. Accordingly, definite proof of the autophagy-inhibitory effect of NBDs in U-2OS cells was obtained by LC3 turnover assay as follows: we performed western blot analysis for LC3-II on lysates from U-2OS cells untreated and NBDs-treated for 24 h, incubated or not with the inhibitor of autophagosomal degradation BAF for 3 h before lysis, as recommended [34] (Fig. 3a). The autophagic flux indexes of the cultures were then calculated as the difference in the amount of LC3-II in the presence and absence of BAF (Fig. 3b), such difference representing the amount of LC3-positive autophagosomes degraded through the lysosome during the last 3 h of culture [28–30]. Of note, in these experiments we chose an endpoint of 24 h, instead of 48 h as in the experiments of Figs. 1 and 2, this in order to assess autophagic flux before the NBDs could induce significant cell death [14]. U-2OS cells treated with NBDs for 24 h had increased levels of LC3-II as compared to the untreated controls (Fig. 3a, black bars of Fig. 3b). Following the addition of BAF the amount of LC3-II was more than doubled in NBD-untreated cultures, whereas it was increased to a much lower degree in NBDs-treated cultures (Fig. 3a, gray bars vs. black bars in Fig. 3b). These results indicate that, albeit with different efficacy, MC3181 and NBDHEX cause an impairment of autophagic flux in U-2OS cells. Indeed, treatment with 5 μM MC3181 or NBDHEX reduced the autophagic flux index of U-2OS cultures from 1 to about 0.4 and 0.2, respectively (Fig. 3c). Figure 3d–f shows the dose-dependent trend in the reduction of the autophagic flux obtained by treating U-2OS cells with NBDHEX in the 0.5–5 μM concentration range.

We next investigated whether, in addition to basal autophagy, the NBDs could impair the increased autophagic flux typical of cells grown under stress conditions, such as for instance under a reduced availability of nutrients. Representative experiments illustrated in Fig. 3g–i demonstrate that the autophagic flux index of cells grown for 24 h in the presence of 0.2 % FBS was about twice that of cells grown in 10 % FBS. Such nutritional stress-induced increase of the autophagic flux was reduced by treatment with MC3181 and completely abolished by NBDHEX.

Overall, these data provide evidence that MC3181 and, to a higher extent, NBDHEX can impair both basal and nutritional stress-induced autophagic flux.

### JNK silencing relieves the impairment of autophagic flux induced by NBDs

**Fig. 4:**
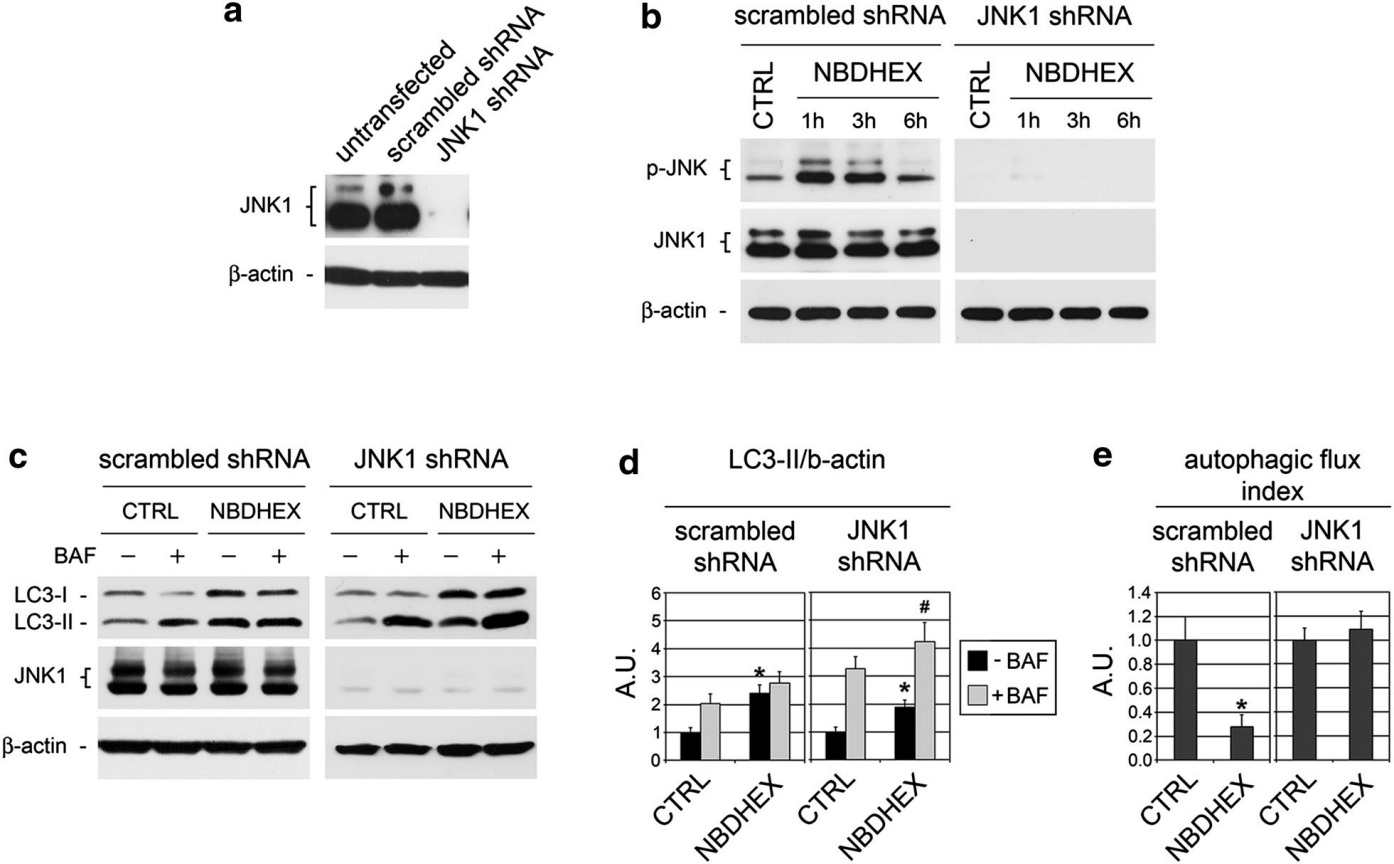
Autophagic flux studies in JNK1-silenced cells. **a** Western blot for JNK1 in untransfected, scrambled shRNA-transfected and JNK1 shRNA-transfected cells. β-Actin was used to ensure equal loading and transfer of samples. **b** Western blot analysis of JNK phospho-activation in cells treated with vehicle (CTRL) or 5 μM NBDHEX for the indicated times. The filter was probed with anti-phospho-JNK (p-JNK) and anti-JNK1 antibodies; β-actin was used to ensure equal loading and transfer of samples. **c** Lysates from scrambled shRNA- and JNK1 shRNA-transfected cells treated with vehicle (CTRL) or 5 μM NBDHEX for 24 h, incubated or not with 100 nmol/l BAF for 3 h before lysis, were subjected to western blot for LC3-II and β-actin. **d** LC3-II/β-actin ratios as determined from the densitometric analysis of autoradiograms obtained in replicates of the experiment shown in **c**, expressed in arbitrary units (mean ± SEM, n ≥ 3; *p < 0.05 vs. CTRL; ^#^p < 0.05 vs. minus BAF). **e** Autophagic flux indexes calculated from the data illustrated in **d** as the difference in LC3-II/actin ratios between samples plus and minus BAF (*p < 0.05 vs. CTRL), expressed in arbitrary units (a.u.)

Previous findings by our group and the results reported hitherto demonstrate that treatment with NBDs triggers the activation of JNK, along with other signaling pathways, and leads to autophagic flux impairment and apoptosis in U-2OS cells [3, 14]. In many studies, however, the activation of JNK by different types of stress signals is reported to induce autophagy [35, 36], with only two recent papers describing JNK as a negative autophagy regulator [37, 38]. Thus, in order to shed light on JNK’s role in the reduction of autophagic flux caused by NBDs, we generated a U-2OS subline with deficient JNK1 expression by stable transfection of the parental cells with an shRNA expression vector. As shown in Fig. 4a, JNK1 was almost undetectable by western blot analysis in the JNK1 shRNA-transfected subclone, whereas in untransfected and scrambled shRNA-transfected U-2OS it was expressed at similar levels. Furthermore, western blot analysis with an anti-pan-phospho-JNK antibody, recognizing both the ubiquitous JNK1 and JNK2 isoforms [36], showed the absence of phospho-active forms of JNK in JNK1-silenced cells treated with NBDHEX while a sustained phospho-activation of JNK was induced by NBDHEX in scramble-transfected cells (Fig. 4b). These findings exclude the possibility that NBDs treatment may lead to the activation of JNK2 in JNK1-silenced U-2OS cells, and demonstrate the functional knockdown of JNK signaling in the silenced clone.

Next, scramble-transfected and JNK1-silenced cells were tested in autophagic flux studies. In particular we performed LC3 turnover assays on cultures treated with NBDHEX, i.e. the compound that showed higher efficacy in reducing the autophagic flux of wild type U-2OS. The effects of NBDHEX on scrambled shRNA cultures were similar to those observed in the parental U-2OS: as compared to the untreated cells, those treated with NBDHEX had an increased level of LC3-II (Fig. 4c, black bars of Fig. 4d) which was only slightly augmented following the addition of BAF (Fig. 4c, gray bars vs. black bars in Fig. 4d). In fact, NBDHEX reduced the autophagic flux index of scramble-transfected cells from 1 to 0.28 (Fig. 4e). On the other hand, in JNK1-silenced cells NBDHEX caused an increase of LC3-II which, following the addition of BAF, was again significantly augmented (Fig. 4c, d). The magnitude of such increase was similar to that obtained after the addition of BAF to NBDHEX-untreated JNK1-silenced cells (Fig. 4c, d). Indeed, NBDHEX caused no net modification of the autophagic flux in the JNK1-deficient subclone (Fig. 4e).

### JNK silencing abolishes caspase-3 activation and apoptosis induced by NBDs

**Fig. 5:**
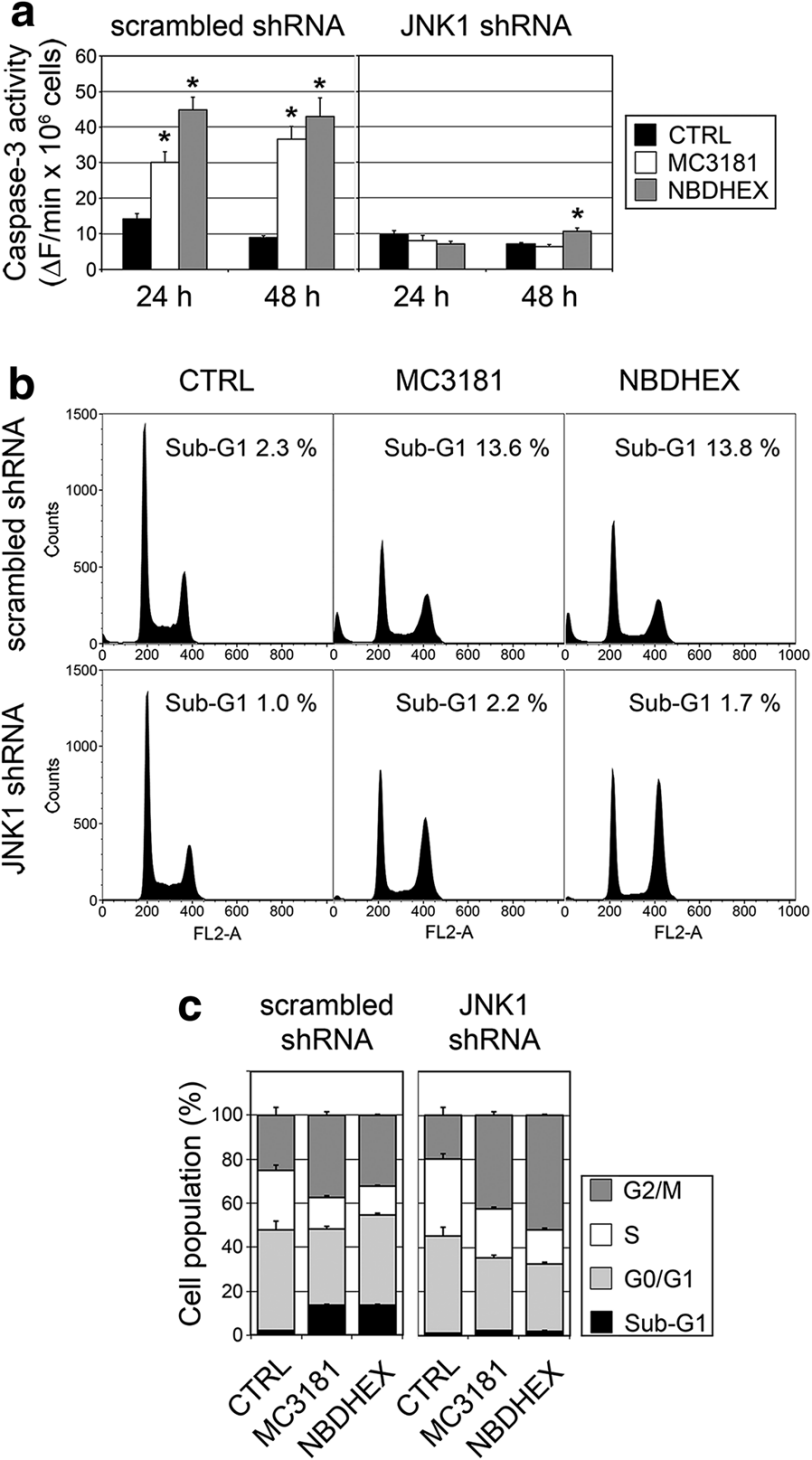
Cell death studies in JNK1-silenced cells. **a** Caspase-3 activity assay in scrambled shRNA- and JNK1 shRNA-transfected cells treated with vehicle (CTRL), 5 μM MC3181 or 5 μM NBDHEX for 24–48 h (mean ± SEM, n ≥ 3; *p < 0.05 vs. CTRL). **b**, **c** Analysis of DNA content by flow cytometry on scrambled shRNA- and JNK1 shRNA-transfected cells treated with vehicle (CTRL), 5 μM MC3181 or 5 μM NBDHEX for 48 h. **b** A representative experiment with the percentage of sub-G1 apoptotic cells is shown. **c** Histograms illustrating the percentage of cells in each phase of cell cycle, resulting from four independent experiments

Finally, JNK1-silenced cells were characterized in terms of sensitivity to the cytotoxic effects of NBDs. By using specific kinase inhibitors, we previously reported that in U-2OS cells NBDHEX causes a p38-dependent arrest in the G2/M phase followed by JNK-dependent apoptosis [3]. Consistent with these findings, the ability of NBDs to induce caspase-3 activity was dramatically compromised in the JNK1-silenced clone (Fig. 5a). Furthermore, concordant results were obtained when evaluating the amount of cells in the sub-G1 phase by flow cytometry. Indeed, scramble-transfected cultures had a percentage of hypodiploid cells of 2.2 ± 0.1 % which was increased to 13.7 ± 0.2 and 13.5 ± 0.4 % by the treatment with MC3181 and NBDHEX, respectively (Fig. 5b, c). Conversely, JNK1-silenced cultures had a proportion of hypodiploid cells of about 1 %, which was only marginally increased by the treatment with either compound (Fig. 5b, c). Besides, the percentage of cells in G2/M arrest following NBDs treatment was much higher in JNK1-silenced cultures than in scramble-transfected cultures (p < 0.05) (Fig. 5b, c). Overall, these findings indicate that in the absence of JNK signaling the apoptotic effect of NBDs was abolished.

## Discussion

Autophagy is an intracellular catabolic pathway by which superfluous or damaged intracellular proteins and organelles are first engulfed by autophagosomes and then delivered to autolysosomes for degradation and recycling of their molecular constituents [39, 40]. Therefore, autophagy is deeply involved in cell homeostasis and adaptation to stress; by promoting the survival of transformed cells under hypoxic, nutritional and therapeutic stress conditions, it plays a major role in the progression of established neoplasms [40, 41]. Cancer cells live under a strong metabolic pressure due to their high energy demand and inefficient energy production; they often reside in a microenvironment that provides an inadequate supply of oxygen, nutrients and growth factors, so that they are predicted to be more susceptible to the suppression of autophagy than non-cancerous cells [29, 39, 40]. On the other hand, prolonged stress and sustained autophagy activation may eventually lead to cell death in certain cellular contexts such as in apoptosis-defective cells [41–43]. Based on such evidence, modulators able to induce autophagic cell death or inhibit protective autophagy are currently being investigated in order to manipulate autophagy for clinical benefit in cancer patients [41–44]. In this context, autophagy inhibition is emerging as a promising therapeutic strategy against cancer [41–44]. Moreover, the potential of autophagy inhibitors such as CQ to increase tumor cell death upon therapeutic stress conditions induced by radiation, genotoxic agents, or tumor-targeted agents is documented by several studies [41–45].

We here demonstrate that NBD compounds, already known to inhibit both the catalytic and TRAF2/JNK1-sequestering activity of GSTs and to trigger cell cycle arrest and apoptosis in cancer cells [4, 5, 14], share the ability to act as late-phase autophagy inhibitors. In fact, we provide evidence that NBDs induce the accumulation of autophagic vesicles and LC3-II while reducing both basal and nutritional stress-induced autophagic flux in U-2OS cells. Furthermore, we show that the effects of NBDs on autophagy are general rather than cell type-specific. Indeed, these compounds produce a variable increase of both LC3-II and the autophagy selective substrate p62 in a panel of tumor cell lines of different origins, the concurrent increase of these markers being consistent with an impairment of autophagosome clearance [27, 28]. At present we do not know whether this effect is due to the inhibition of autophagosome-lysosome fusion or to the defective degradation of the autophagic material in the autophagolysosome [27].

The tumor cell lines used in this study display different degrees of sensitivity to the cytostatic/cytotoxic effects of NBDHEX and MC3181, their IC_50_s for the two compounds ranging from about 1 μM for U-2OS to about 11–16 μM for HT-29 cells. No evident correlation emerges from the comparison of the cell lines’ sensitivity to the cytostatic/cytotoxic effects of NBDs and the degree of autophagy inhibition induced by the compounds. For instance, based on autophagic markers accumulation levels, NBDs induce comparably high levels of autophagy impairment in U-2OS and HT-29 cells, which however display the lowest and highest IC_50_ values among the cell lines included in the study. Conversely, the IC_50_s of U-2OS, MM-B1 and MCF-7 cells are quite similar, while the extent of autophagy inhibition observed in the latter two cell lines appears much lower as compared to that of U-2OS. On the other hand, this apparent lack of correlation is not conclusive since different cell lines can be characterized by different degrees of autophagy addiction and, accordingly, can display marked differences in how they respond to autophagy inhibition [29, 44, 46, 47]. Therefore, experiments performed in a given cell line after silencing of essential autophagy genes [27] will be necessary to investigate whether and to what extent the effects of NBDs on tumor growth and survival actually depend on the impairment of the autophagic pathway. In any case, our results suggest that the therapeutic potential of NBDs may not rely solely on their effectiveness in inducing cell cycle arrest and apoptosis, but also on their ability to weaken the capacity of tumor cells to endure stress conditions via autophagy. These findings may bear relevance for future studies specifically aimed at evaluating the efficacy of NBDs on autophagy-dependent tumor types as well as for the rational design of combined approaches based on the association of NBDs with antitumor drugs known to induce pro-survival autophagy [43–45].

A second interesting finding of this study regards the role played by JNK in the autophagy-inhibitory effect of NBDs. In fact, treatment with NBDs triggers the activation of JNK, along with other signaling pathways [3, 5, 14, 21], and leads to autophagic flux impairment. Moreover, by silencing JNK1 expression in U-2OS cells we here provide evidence that autophagy impairment by NBDs requires JNK activity: as compared to the untreated cells, in JNK1-silenced cells treated with NBDHEX we found an increase of LC3-II compatible with an increased formation of autophagosomes, but autophagic flux inhibition was no longer observed. These results implying a role for JNK in mediating autophagy impairment are unexpected given that the literature extensively supports the pro-autophagic function of this MAPK [35, 36]. In particular, JNK has been reported to promote autophagy in response to different types of stress signals by two main mechanisms. First, by phosphorylating Bcl-2, JNK induces its dissociation from the autophagy-regulatory protein Beclin-1 which, in turn, interacts with multiple partners to promote the formation and maturation of autophagosomes [36]. Second, JNK activation drives the upregulation of damage-regulated autophagy modulator (DRAM), a lysosomal protein whose stimulatory role in autophagy is thought to rely on the ability to regulate the fusion of autophagosomes and lysosomes [48]. By contrast, two recent papers report that suppression of JNK signaling induces autophagy in neurons [37], lens fiber cells and MCF-7 cells [38] by decreasing FoxO-dependent expression of Bnip3, which in turn promotes autophagy via the dissociation of Beclin1 from Bcl-xl [37], and by acting as a positive regulator of the autophagy inhibitor MTORC1 [38]. Therefore, it appears that the autophagy inhibitory action of JNK can be mediated by different mechanisms. Besides, a factor that may explain the involvement of JNK in the inhibition of autophagy caused by NBDs is the JNK-dependent activation of caspases, since it has been reported that these proteases can inhibit autophagy through the cleavage of essential autophagy proteins including Beclin-1 and different Atg proteins [49]. The finding that NBDs caused autophagy impairment and caspase-3 activation in JNK-positive U-2OS, but no autophagic flux inhibition or caspase-3 activation in JNK-silenced cells supports this hypothesis.

What emerges from this complex scenario is that JNK can act at multiple levels in the dynamic multistep process of autophagy to generate context-specific responses, probably depending on the mode and kinetics of its activation as well as on the cooperation with different signal transduction pathways [37]. In this respect, due to their ability to induce the dissociation of both the TRAF2-GSTP1-1 and JNK-GSTP1-1 complexes, NBDs act as multi-target compounds able to activate not only JNK but also the TRAF2 downstream target p38 [3, 14, 21], which has been reported to act, through mechanisms still poorly defined, both as a positive and negative regulator of autophagy [35, 36]. Accordingly, the concurrent activation of different signaling pathways by NBDs may participate in modulating JNK-dependent responses leading to autophagic flux impairment.

## Conclusions

This study provides further evidence for the concept that, in addition to its well-established role as a positive autophagy regulator, JNK can participate in impairing autophagy in certain conditions. Furthermore, our demonstration that NBDs can act as late-phase autophagy inhibitors unravels an additional favorable property of these compounds and opens up new opportunities to fully exploit their potential as anticancer agents.

## Abbreviations

BAF: bafilomycin A1
CQ: chloroquine diphosphate
GST: glutathione transferase
GSTP1-1: P1-1 isoform of GST
JNK: c-Jun N-terminal kinase
MAPK: mitogen-activated protein kinase
MC3181: 2-(2-(2-((7-nitrobenzo[c][1,2,5]oxadiazol-4-yl)thio)ethoxy)ethoxy)ethanol
NBD: nitrobenzoxadiazoles
NBDHEX: 6-((7-nitrobenzo[c][1,2,5]oxadiazol-4-yl)thio)hexan-1-ol
PLA: proximity ligation assay
TRAF2: TNF-receptor associated factor 2
